# Effect of miR-499-5p/SOX6 axis on atrial fibrosis in rats with atrial fibrillation

**DOI:** 10.1515/med-2023-0654

**Published:** 2023-05-12

**Authors:** Xinyuan Han, Shunda Wang, Zhijun Yong, Xueting Zhang, Xuanqi Wang, Penghua You

**Affiliations:** Department of Rehabilitation Medicine, Shaanxi Provincial People’s Hospital, Xi’an, Shaanxi, 710068, China; Department of Cardiology, Shaanxi Provincial People’s Hospital, No. 256 Youyi West Road, Xi’an, Shaanxi, 710068, China

**Keywords:** atrial fibrillation, atrial fibrosis, miR-499-5p, SOX6, p21, cell senescence, apoptosis, microRNA

## Abstract

Atrial fibrosis is involved in the progression of atrial fibrillation (AF). miR-499-5p is the most downregulated microRNA in arrhythmogenic cardiomyopathy hearts. Sry-related high-mobility-group box 6 (SOX6) is associated with apoptosis, inflammatory responses, and fibrosis. This study investigated the mechanism of miR-499-5p in ameliorating AF rats by regulating SOX6. AF rat models were established by injecting Ach–CaCl_2_ mixture, and the rats were treated with Lv-miR-499-5p/oe-SOX6/si-SOX6 before modeling. AF duration was recorded using electrocardiogram. miR-499-5p and SOX6 expression levels in the myocardium were determined by reverse transcription-quantitative polymerase chain reaction. The binding of miR-499-5p and SOX6 was validated. The atrial fibrosis degree and cardiomyocyte apoptosis were assessed using the Masson and terminal deoxynucleotidyl transferase-mediated dUTP nick-end labeling staining methods. Levels of SOX6, atrial fibrosis markers (collage I/α-SMA/TGFβ1), cell cycle-related proteins (p21/CDC25/Cyclin B1), and cell senescence markers (SA-β-gal/γ-H2AX) were measured using Western blotting and immunohistochemistry. miR-499-5p was downregulated and SOX6 was upregulated in AF rats. miR-499-5p overexpression shortened the AF duration, alleviated atrial fibrosis, and decreased collage I/α-SMA/TGFβ1. miR-499-5p targeted SOX6 to ameliorate atrial fibrosis. AF rats exhibited increased p21/CDC25/Cyclin B1/SA-β-gal/γ-H2AX levels and raised cardiomyocyte apoptosis. SOX6 silencing downregulated p21 and alleviated cardiomyocyte cycle arrest, cell senescence, and apoptosis in AF rats. Shortly, miR-499-5p suppresses atrial fibrosis and cardiomyocyte senescence by targeting SOX6 and downregulating p21, thus mitigating AF in rats.

## Graphical abstract

**Figure:**
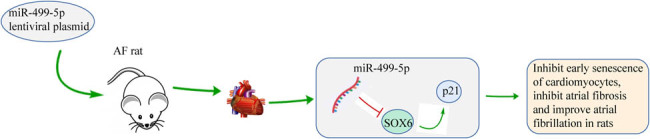

## 1 Introduction

Atrial fibrillation (AF) is a complicated cardiomyopathy based on arrhythmia substrates, which undermines the quality of life and functional status and increases mortality due to atrioventricular dyssynchrony, altered hemodynamics, progressive atrial and ventricular mechanical dysfunction, and thromboembolic complications [1,2]. AF roughly affects more than 33 million people worldwide, and its attack rate increases with age, with an estimated lifetime risk exceeding 30% [3]. In mechanism, atrial remodeling occupies the central link in AF pathogenesis, and atrial fibrosis, the most pivotal pathological change of atrial remodeling, severely disrupts the continuity of myocardial electrical conduction, results in local conduction disorders, and promotes the occurrence and maintenance of AF, making its degree a paramount indicator for evaluating AF [4,5]. Intrinsically, atrial fibrosis consists of numerous individual and multifactorial processes caused by complicated interactions between various neurohormonal and cellular mediators and is manifested in response to diverse cardiac injurious stimuli, which is featured primarily by the enormous deposition of extracellular matrix [6,7]. Consequently, novel and effective molecules targeting atrial fibrosis are of considerable importance for the treatment and management of AF.

microRNAs (miRNAs) are short, single-stranded RNAs with roughly 22 nucleotides in length that anneal with sequences primarily located in 3′-UTR of mRNA, which contribute to mRNA degradation and translation inhibition to suppress protein expression, and exert prominent roles in cardiovascular disorders [8]. miRNAs emerge as potent biomarkers for AF diagnosis owing to their strong stability and easy availability in atrial tissues and circulating blood, and they are exceedingly involved in AF etiology by regulating atrial remodeling and atrial fibrosis [9,10]. Interestingly, a transcriptomic analysis of atrial samples from human AF individuals has uncovered the 2-fold upregulation of miR-223, miR-328, and miR-664, but at least 50% downregulation of miR-499 [11]. Calore et al. also have reported that miR-708-5p and miR-217-5p are the most upregulated miRNAs, whereas miR-499-5p is the most downregulated miRNA in arrhythmogenic cardiomyopathy hearts [12]. More importantly, the imperative role of miR-499-5p in fibrosis is unveiled: for instance, Wharton’s jelly-derived mesenchymal stem cells can reduce fibrosis in mouse models of Duchenne muscular dystrophy by upregulating miR-499-5p [13]. miR-499-5p can mitigate pulmonary fibrosis in mice with sepsis-induced lung injury by targeting Sry-related high-mobility-group box 6 (SOX6) [14]. Additionally, there is evidence to illustrate that a myriad of SOX proteins, especially SOX6, is implicated in heart functions [15,16,17]. From the aforementioned findings, it is reasonable to speculate that miR-499-5p may affect AF and regulate atrial fibrosis through SOX6.

It is noteworthy that cardiomyocyte senescence represents a vital contributor to AF and facilitates detrimental atrial remodeling during AF progression [18]. Cell senescence is mainly characterized by increased levels of cell cycle inhibitors p16, p21, and p53 [19,20]. The upregulation of p21 is noted in chronic AF individuals, and p21 is also tightly associated with cardiomyocyte apoptosis [21]. Of utmost interest, previous research has documented an association between p21 and SOX6 in tumors [22,23]. However, the interaction between SOX6 and p21 in AF remains largely an unknown domain. Therefore, with AF rats as the animal models, this study probed into the action of miR-499-5p/SOX6 axis in atrial fibrosis through p21, hoping to provide paramount reference values for AF therapy.

## 2 Materials and methods

### 2.1 AF rat modeling and grouping

A total of 48 male Sprague–Dawley rats (weighing 230–250 g) purchased from Vital River (SYXK [Beijing] 2016-0011, Beijing, China) were reared at 22–26°C under 40–60% humidity, with 12 h/12 h light/dark cycles and *ad libitum* to food and water.

Randomly, 42 rats were selected to establish AF rat models: the rats were subjected to a daily injection of 1 mL/kg mixed solution of acetylcholine (Ach; 60 μL/mL, B50001, leaf source creatures; Beijing Wuyejia Technology, Beijing, China) and CaCl_2_ (10 mg/mL) through tail veins for 7 days. The presence of standard f-waves and the absence of P-waves during an electrocardiogram (ECG) test can reflect the successful modeling of AF animals [24,25,26]. The remaining six rats were injected with 1 mL/kg normal saline daily via tail veins for 7 days and served as the control group.

The 42 AF rats were randomized to the following seven groups, with six rats per group: (1) AF group; (2) AF + Lv-miR group: subjected to a single injection of lentivirus (Lv)-miR-499-5p [27]; (3) AF + Lv-negative control (NC) group: subjected to a single injection of Lv-NC; (4) AF + Lv-miR + oe-SOX6 group: subjected to a single and simultaneous injection of Lv-miR-499-5p and lentiviral vector-constructed SOX6 overexpression plasmid oe-SOX6; (5) AF + Lv-miR + oe-NC group: subjected to a single and simultaneous injection of Lv-miR-499-5p and oe-NC; (6) AF + si-SOX6 group: subjected to a single injection of SOX6 interference plasmid si-SOX6; and (7) AF + si-NC group: subjected to a single injection of lentiviral vector-constructed si-NC. Lv-miR-499-5p, Lv-NC, oe-SOX6, oe-NC, si-SOX6, and si-NC plasmids were designed and tested for quality by Bio Scientific (Shanghai, China) and injected into rats through the tail vein (all at 2 × 10^11^ plasmids/rat) at day 14 prior to AF modeling.

After experimentation, AF state in rats was evaluated through ECG. Afterward, experimental rats were euthanized with an intraperitoneal injection of 150 mg/kg of 2% pentobarbital sodium, and the hearts were rapidly removed, rinsed with phosphate-buffered saline (PBS), and preserved in a −80°C freezer for subsequent usage.

### 2.2 ECG recording and analyzing

Rats were anesthetized with 40 mg/kg pentobarbital sodium. Subsequently, the ECG of the rats was recorded with a standard lead II utilizing the MedLab-U/4C501H biological signal collection system. After the injection of Ach–CaCl_2_ mixed solution for 7 days, the typical AF ECG showing the absence of P-waves and presence of f-waves was considered the sign of AF occurrence, while the restoration of sinus rhythm, presence of P-waves, and absence of f-waves indicated the AF termination, with the period from occurrence to termination as the duration of AF. The induction time and duration of AF were recorded.

### 2.3 Reverse transcription-quantitative polymerase chain reaction (RT-qPCR)

Total RNA from rat myocardial tissues was extracted with TRIzol reagents (Invitrogen, Carlsbad, CA, USA). After measuring the RNA concentration with NanoDrop 2000 (Thermo Fisher Scientific, Waltham, MA, USA), cDNA synthesis and reaction were performed in a polymerase chain reaction (PCR) amplifier (Thermo Fisher Scientific). RT-qPCR was processed using an ABI7500 qPCR instrument (Thermo Fisher Scientific) under the following conditions: pre-denaturation at 95°C for 10 min and then 45 PCR cycles of denaturation at 95°C for 15 s, annealing at 60°C for 1 min, and extension at 72°C for 10 s. Primers are listed in Table 1 [28,29]. Quantification was performed with the 2^−ΔΔCt^ method [30], with the relative expression normalized to U6 or glyceraldehyde-3-phosphate dehydrogenase (GAPDH).

**Table 1:** Primer sequences

Gene	Forward (5′-3′)	Reverse (5′-3′)
*miR-499-5p*	TTAAGACTTGCAGTGATGTTT	GTGCAGGGTCCGAGGT
*U6*	TAAAATCTATACACGACGGCTTCG	TACTGTGCGTTTAAGCACTTCGC
*SOX6*	CCCCTCTGAACATGGTGGTGGC	TGAGACTGCCCCTGCCGAGT
*GAPDH*	TCTCCCTCACAATTTCCATCCC	TTTTTGTGGGTGCAGCGAAC

### 2.4 Western blotting

Total protein was extracted from heart tissue using radioimmunoprecipitation assay lysis buffer (Millipore Corporation, Billerica, MA, USA), and total protein concentration in tissues was determined using bicinchoninic acid kits (Beyotime, Shanghai, China). Protein lysate was separated by sodium dodecyl sulfate–polyacrylamide gel electrophoresis and later transferred onto polyvinylidene fluoride membranes. After 2 h of storage in 5% skim milk, membranes were probed overnight with primary antibodies such as anti-SOX6 (1:500, ab64946; Abcam, Cambridge, MA, USA), anti-collage I (1:1,000, ab138492; Abcam), anti-α-smooth muscle actin (α-SMA; 1:1,000, ab108424; Abcam), anti-transforming growth factor-β1 (TGFβ1; 0.5 μg/mL, ab92486; Abcam), anti-p21 (ab109199, 1/1,000; Abcam), anti-cell division cycle 25 (CDC25; 1:1,000, ab111830; Abcam), and anti-Cyclin B1 (1:200, ab215436; Abcam). Following washing with Tris-buffered saline/Tween 20, the membranes were then incubated with secondary antibody goat anti-rabbit IgG H&L horse radish peroxidase (1:2,000, ab205718; Abcam) for 1 h. Immunoreactive bands were subsequently visualized by enhanced chemiluminescence and later quantified through densitometric analysis (Quantity One; Bio-Rad, Hercules, CA, USA), with GAPDH (1:10,000, ab8245; Abcam) as an internal control.

### 2.5 Masson staining

The hearts were cut into coronal plane segments, fixed with 4% paraformaldehyde at 4°C, then dried in graded ethanol, and paraffin-embedded, followed by cutting into 4 μm thick sections. The sections were stained with Masson’s trichrome and subsequently imaged using a digital camera on a microscope (Olympus, Tokyo, Japan). ImageJ software (NIH, Bethesda, MD, USA) was used to quantify the percentage of blue-positive stained areas to the total tissue area, to reflect the degree of fibrosis.

### 2.6 Dual luciferase assay

The targeted binding sites of miR-499-5p and SOX6 3′-UTR were predicted via TargerScan7.2 database (http://www.targetscan.org/vert_72/). HEK293T cells at the exponential phase were put in 96-well plates. When reaching 70% confluence, the cells were subjected to transfection using Lipofectamine 2000. The SOX6-wild type (WT) and SOX6-mutant (MUT) plasmids (GenePharma, Shanghai, China) were co-transfected into HEK293T cells with mimics NC and miR-499-5p mimics (GenePharma), respectively. After 48 h, the cells were collected and lysed, followed by the detection of luciferase activity using luciferase assay kits (BioVision, San Francisco, CA, USA) and a Glomax 20/20 luminometer (Promega, Madison, WI, USA).

### 2.7 Immunohistochemistry

The paraffin-embedded myocardial tissues were cut into 4 μm thick sections, deparaffinized with xylene (Sigma-Aldrich, Merck KGaA, Darmstadt, Germany), and next treated with 3% H_2_O_2_ for quenching endogenous peroxidase actions. Following incubation with citrate buffer for 30 min in a steamer, the sections were stained with primary antibodies anti-senescence-associated β-galactosidase (SA-β-gal; 1:500, ab203749; Abcam) and anti-γ-histone 2AX (γ-H2AX; 1:500, ab124781; Abcam) overnight at 4°C, and later with secondary antibody goat anti-rabbit IgG (1:1,000, ab6721; Abcam) for 2 h. Subsequently, the sections were stained again with diaminobenzidine and then counterstained with hematoxylin. With rabbit anti-IgG (1:500, ab172730; Abcam) as an isotype control, the sections were scrutinized under a microscope (Olympus), and levels of SA-β-gal and γ-H2AX were measured using Image J software (NIH).

### 2.8 Terminal deoxynucleotidyl transferase-mediated dUTP nick-end labeling (TUNEL) staining

The paraffin-embedded myocardial tissues were cut into 3 μm thick sections, dehydrated through routine deparaffinization, and next incubated with PBS containing 10% fetal bovine serum (SND-X0108; Sinoda Biotechnology, Nanjing, Jiangsu, China) for 30 min. Following addition with 50 μL of TUNEL reaction mixture (Roche, Basel, Switzerland), the sections were incubated thrice in a wet box at 37°C and subsequently supplemented with 50 μL of conversion agent peroxidase (Roche), followed by another 30 min incubation in the wet box. The sections were supplemented with diaminobenzidine reagent, followed by the observation of color development status under a microscope. After stopping the color development by adding water, the sections were placed in hematoxylin for 2 min and successively immersed in 95% ethanol I–II, in anhydrous ethanol I–II for 3–5 min, and in xylene I–II for 3–5 min. Subsequently, the sections were mounted with neutral gel and observed under a fluorescence microscope (Olympus).

### 2.9 Statistical analysis

Data analyses and plotting were processed using GraphPad Prism 8.0.1 (GraphPad Software Inc., San Diego, CA, USA). Measurement data were described as mean ± standard deviation. The *t*-test was conducted for comparisons between two groups, with one-way analysis of variance (ANOVA) for multiple groups. Tukey’s multiple comparison test was used for the *post hoc* test. The *p* value was acquired by a two-sided test, and *p* < 0.05 indicated statistical significance.


**Ethics approval and consent to participate:** All animal experiments were conducted in concert with the instructions of Animal Ethics Committee of Shaanxi Provincial People s Hospital. Significant efforts were made to minimize the animal number and their suffering. The animal experiments have been carried out in accordance with the ARRIVE guidelines.

## 3 Results

### 3.1 miR-499-5p was underexpressed and SOX6 was upregulated in myocardial tissues of AF rats

**Figure 1:**
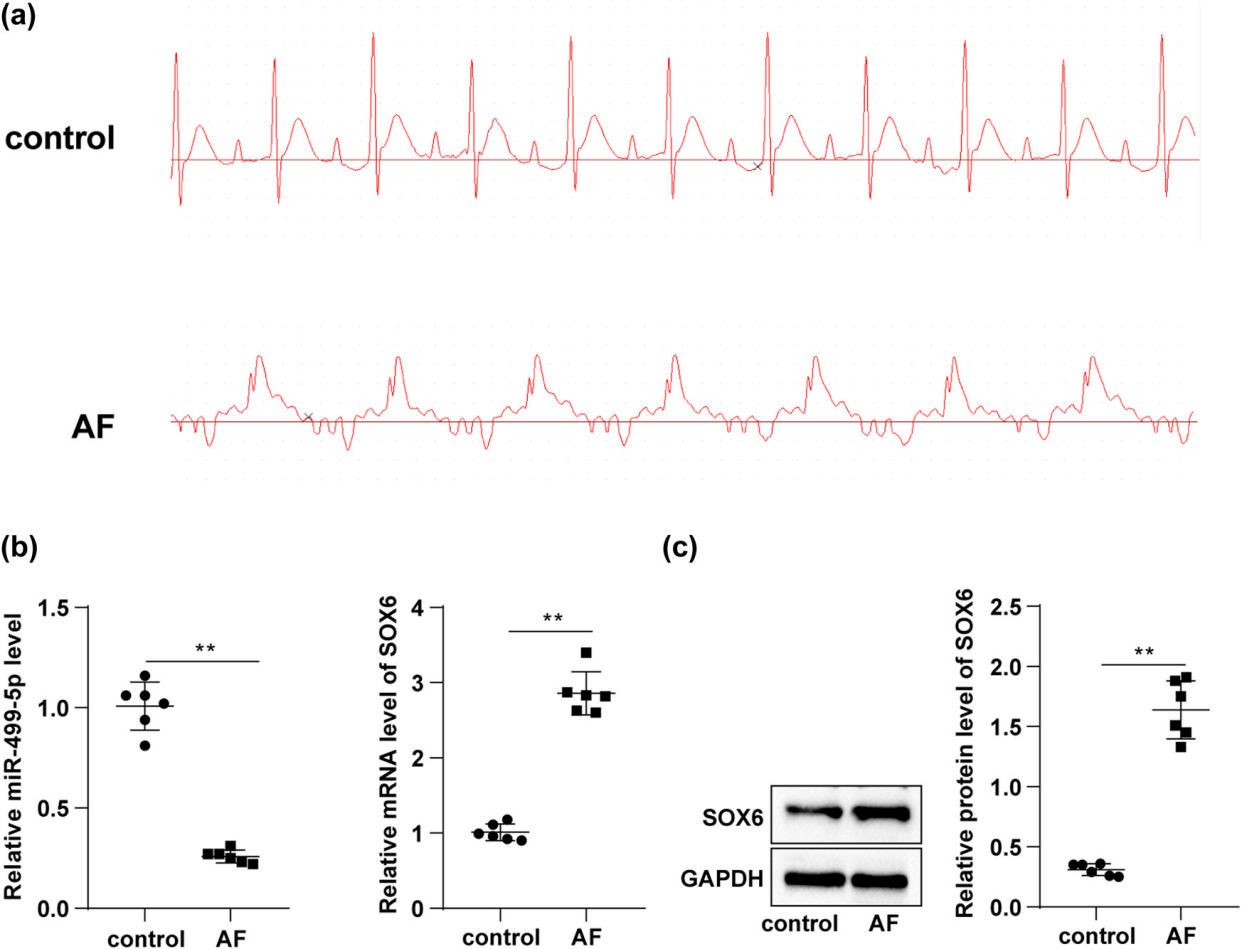
miR-499-5p was underexpressed in myocardial tissues of AF rats and inversely correlated with SOX6. Rats were injected daily with Ach–CaCl_2_ mixture via tail veins for 7 days. (a) ECG analysis; (b) RT-qPCR determined the levels of miR-499-5p and SOX6 in rat myocardial tissues; (c) Western blotting determined the SOX6 protein levels in rat myocardial tissues. *N* = 6. An independent sample *t*-test was used for comparisons between groups in panels b and c. **p < 0.01.

We first injected the Ach–CaCl_2_ mixture into rats daily via tail veins for 7 days. The ECG analysis showed the regular P-waves in the control group (indicating normality of sinus rhythm), but the absence of P-waves and the presence of irregular R–R intervals in the AF group, with the induction time and duration of AF of (4.79 ± 0.62) s and (6.72 ± 0.68) s, respectively (Figure 1a), suggesting that Ach–CaCl_2_ mixture successfully induced AF in rats. Subsequently, the levels of miR-499-5p and SOX6 in myocardial tissues were determined using RT-qPCR, which indicated that miR-499-5p was notably downregulated and SOX6 was prominently upregulated in the AF group compared with the control group (Figure 1b, *p* < 0.01). Western blotting revealed that the AF group had a higher SOX6 protein level in rat myocardial tissues than the control group (Figure 1c, *p* < 0.01). The aforementioned results evinced that miR-499-5p was weakly expressed and SOX6 was highly expressed in myocardial tissues of AF rats.

### 3.2 miR-499-5p alleviated AF in rats by inhibiting atrial fibrosis

**Figure 2:**
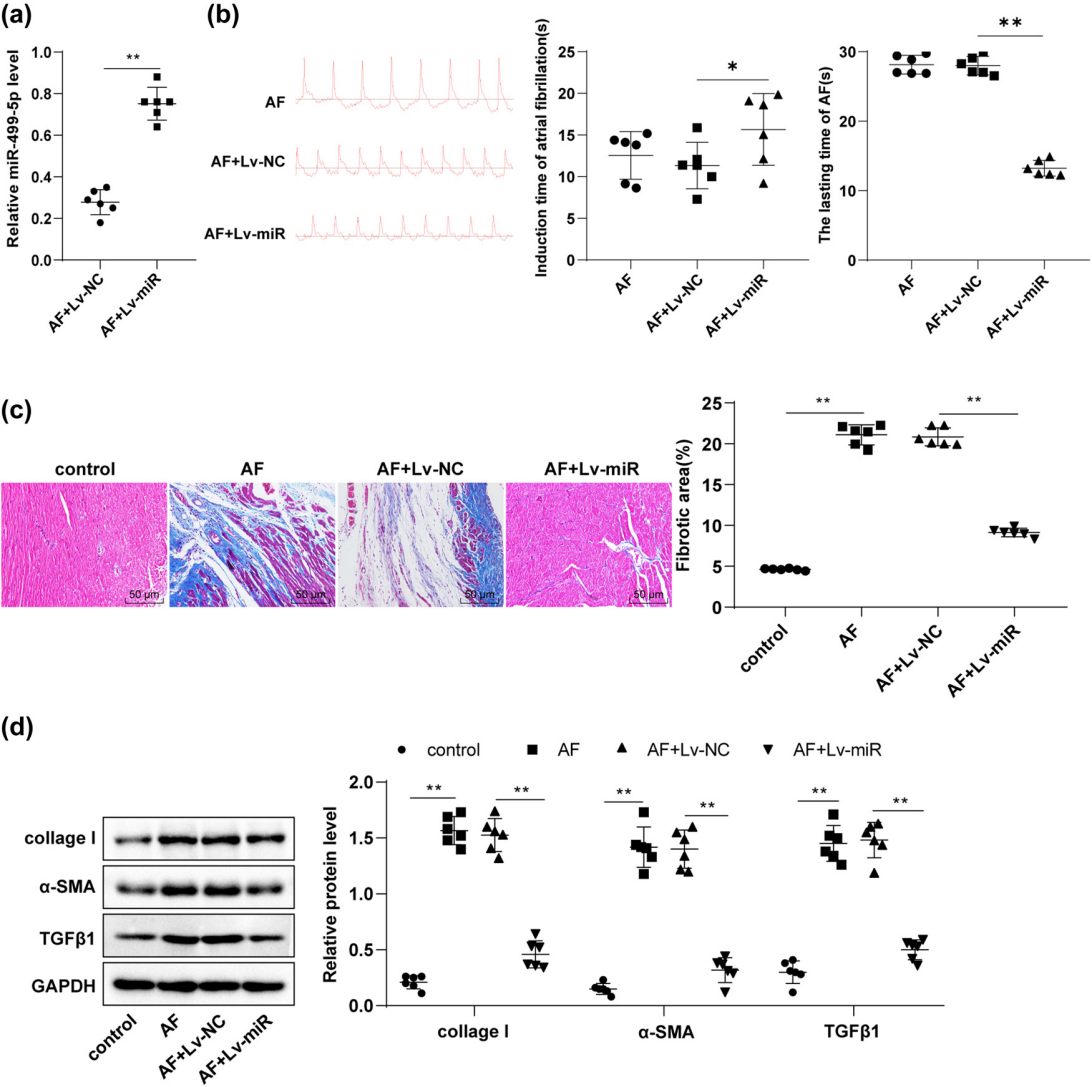
miR-499-5p alleviated AF in rats by inhibiting atrial fibrosis. Rats were injected with Lv-miR-499-5p/Lv-NC plasmids via tail veins, followed by AF modeling 14 days later. (a) RT-qPCR measured miR-499-5p level in rat myocardial tissues; (b) ECG analysis; (c) Masson staining evaluated atrial fibrosis in rats; (d) Western blotting measured the protein levels of atrial fibrosis markers collage I, α-SMA, and TGFβ1; *N* = 6. An independent sample *t*-test was used for comparisons between two groups in panel (a), and one-way ANOVA was used for comparisons among multiple groups in panels (b)/(c)/(d), followed by Tukey’s test. *p < 0.05, **p < 0.01.

To further investigate the roles of miR-499-5p in AF rats, Lv-miR-499-5p or Lv-NC plasmids were injected into rats via the tail vein, followed by AF modeling 14 days later. RT-qPCR revealed the significant upregulation of miR-499-5p in myocardial tissues of the AF + Lv-miR group relative to the AF + Lv-NC group (Figure 2a, *p* < 0.01), indicating the successful transfection of Lv-miR-499-5p plasmids. Subsequently, ECG analysis suggested that the AF + Lv-miR group has a longer induction time of AF and a shorter duration than the AF + Lv-NC group (Figure 2b, *p* < 0.05). It is fully acknowledged that atrial fibrosis is the most paramount pathological alternation of atrial remodeling and is a vital indicator for assessing AF development [4,5], so we further used the Masson staining to evaluate the atrial fibrosis in rats. As indicated, the control rats had normal amounts of collagen fibers and dark-red cytoplasm and muscle fibers, but no punctate or patchy necrosis in the atrial myocardium interstitium; AF rats exhibited large areas of confluent necrosis, abnormally proliferated collagen fibers, and high degree and extended areas of atrial fibrosis; however, the injection of Lv-miR-499-5p plasmids prominently alleviated the fibrosis in AF rats (Figure 2c, *p* < 0.01). Western blotting unraveled that AF rats presented higher protein levels of atrial fibrosis markers such as collage I, α-SMA, and TGFβ1 in myocardial tissues than control rats; however, treatment with Lv-miR-499-5p plasmids noticeably reduced the collage I, α-SMA, and TGFβ1 levels in AF rats (Figure 2d, *p* < 0.01). Altogether, miR-499-5p repressed atrial fibrosis to mitigate AF in rats.

### 3.3 miR-499-5p attenuated atrial fibrosis by targeting SOX6 in AF rats

**Figure 3:**
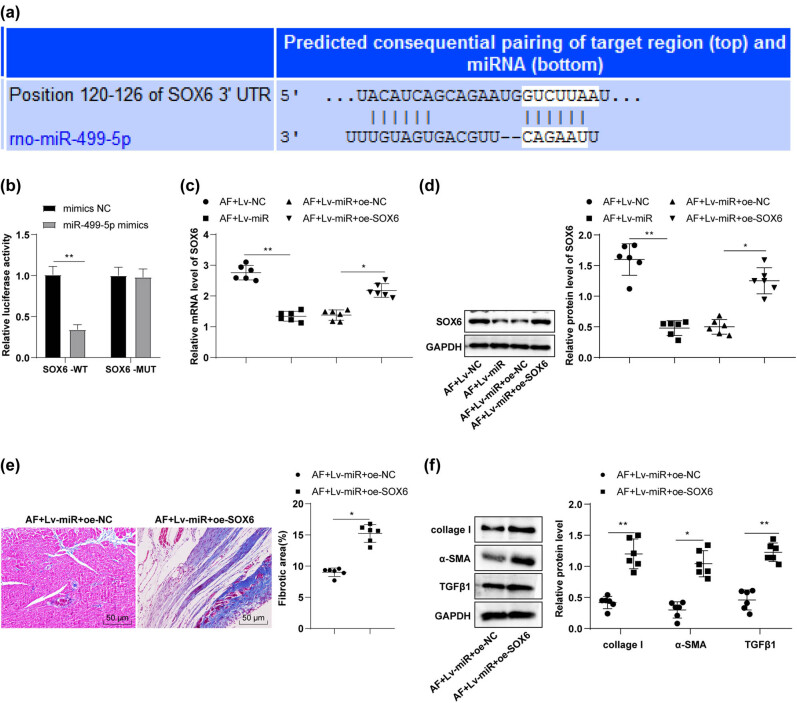
miR-499-5p attenuated atrial fibrosis by targeting SOX6 in AF rats. (a) TargerScan7.2 database predicted the targeted binding sites of miR-499-5p and SOX6; (b) the dual-luciferase assay; (c) RT-qPCR determined SOX6 mRNA level in rat myocardial tissues; (d) Western blotting determined SOX6 protein level in rat myocardial tissues; (e) Masson staining evaluated atrial fibrosis in rats; (f) Western blotting determined collage I, α-SMA, and TGFβ1 protein levels in rat myocardial tissues. *N* = 6. An independent sample *t*-test was conducted for comparisons between groups in panels (b)/(e)/(f), and one-way ANOVA was used for multiple groups in panels c/d, followed by Tukey’s test. *p < 0.05, **p < 0.01.

Based on previous studies, we speculated that miR-499-5p might relieve atrial fibrosis in AF rats by targeting SOX6 expression. To validate the speculation, the targeted binding sites of miR-499-5p and SOX6 were predicted using TargerScan7.2 (http://www.targetscan.org/vert_72/) (Figure 3a) and subsequently verified through a dual luciferase assay. The results unveiled that the cellular luciferase activity was decreased after concomitant transfection of miR-499-5p mimics and SOX6-WT plasmids (*p* < 0.01), but showed no significant difference after concomitant transfection of miR-499-5p mimics and SOX6-MUT plasmids (Figure 3b, *p* > 0.05), indicative of the targeted binding relationship between miR-499-5p and SOX6. Furthermore, RT-qPCR and Western blotting revealed reduced SOX6 levels in the AF + Lv-miR group compared with the AF + Lv-NC group (Figure 3c and d, *p* < 0.01), suggesting that miR-499-5p targeted and inhibited SOX6 expression. Finally, Lv-miR-499-5p and lentiviral vector-constructed oe-SOX6 plasmids were concomitantly injected into rats via the tail vein, followed by AF modeling 14 days later. Relative to the AF + Lv-miR + oe-NC group, the AF + Lv-miR + oe-SOX6 group had increased SOX6 levels (Figure 3c and d, *p* < 0.01); aggravated atrial fibrosis (Figure 3e, *p* < 0.05); and raised collage I, α-SMA, and TGFβ1 protein levels in myocardial tissues (Figure 3f, *p* < 0.05). In summary, miR-499-5p mitigated atrial fibrosis in AF rats by targeting SOX6.

### 3.4 AF upregulated p21 to trigger cell cycle arrest, senescence, and apoptosis in rat cardiomyocytes

**Figure 4:**
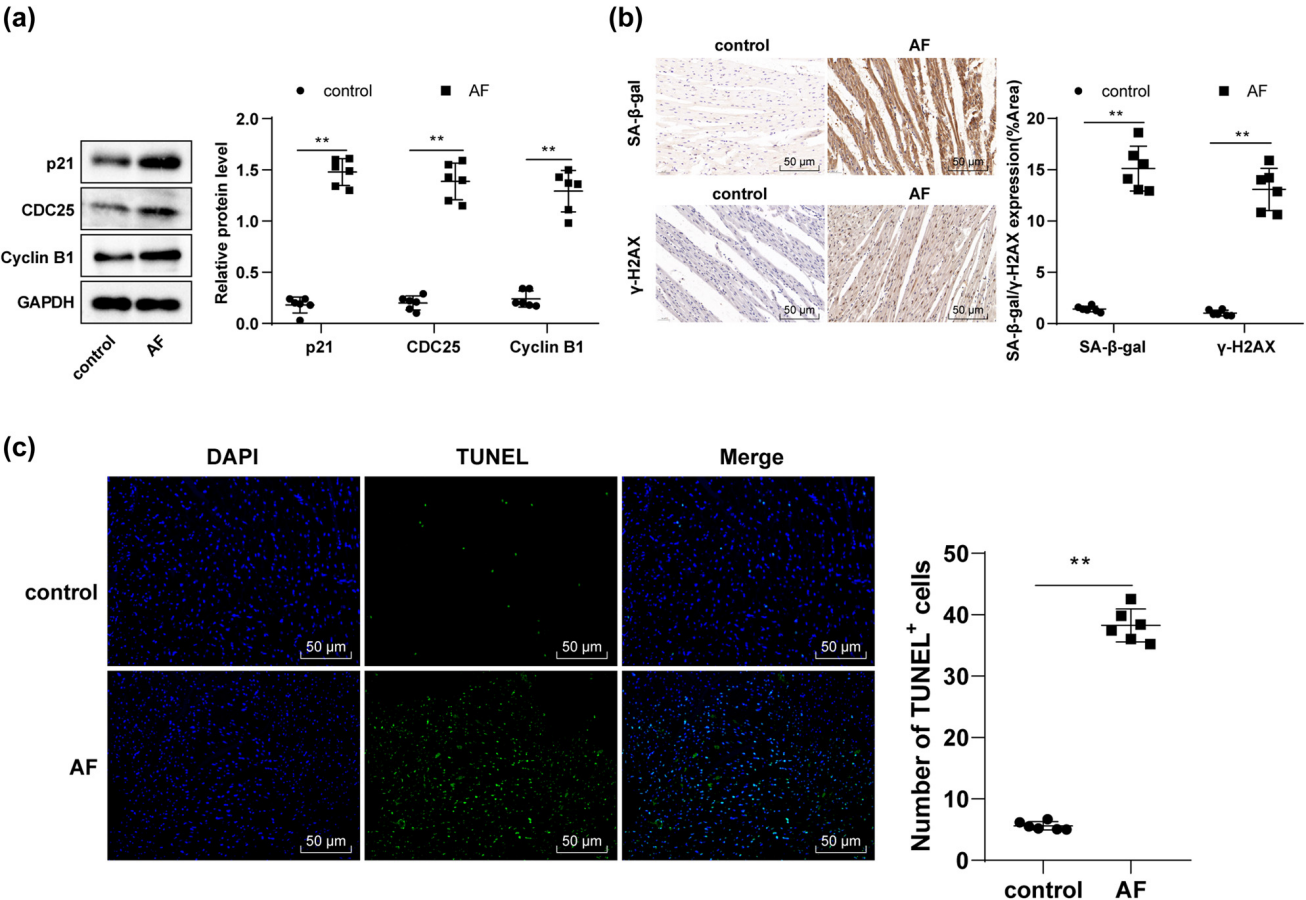
AF upregulated p21 to trigger cell cycle arrest, senescence, and apoptosis in rat cardiomyocytes. (a) Western blotting determined p21, CDC25, and Cyclin B1 protein levels in myocardial tissues; (b) immunohistochemistry assay determined SA-β-gal and γ-H2AX levels in myocardial tissues; (c) TUNEL staining evaluated cell apoptosis. *N* = 6. An independent sample *t*-test was used for comparisons between two groups. **p < 0.01.

Previous research has evinced the close association between p21-dependent G2 cell cycle arrest and fibrotic diseases [31]. In addition, the upregulation of cyclin-dependent kinase inhibitors p21 and p16 is sufficient to promote premature aging and senescence of atrial endothelial cells [32]. Therefore, we conducted Western blotting to determine the levels of cyclin proteins in myocardial tissues and found raised protein levels of p21, CDC25, and Cyclin B1 in AF rats (Figure 4a, *p* < 0.01). Immunohistochemistry assay illustrated that AF rats had higher levels of SA-β-gal and γ-H2AX (markers of cell senescence) in myocardial tissues than control rats (Figure 4b, *p* < 0.01). TUNEL staining revealed increased cardiomyocyte apoptosis in AF rats compared with control rats (Figure 4c, *p* < 0.01). Briefly, AF upregulated p21 levels to facilitate cell cycle arrest, senescence, and apoptosis in rat cardiomyocytes.

### 3.5 SOX6 silencing downregulated p21 and mitigated cell cycle arrest, senescence, and apoptosis in rat cardiomyocytes

**Figure 5:**
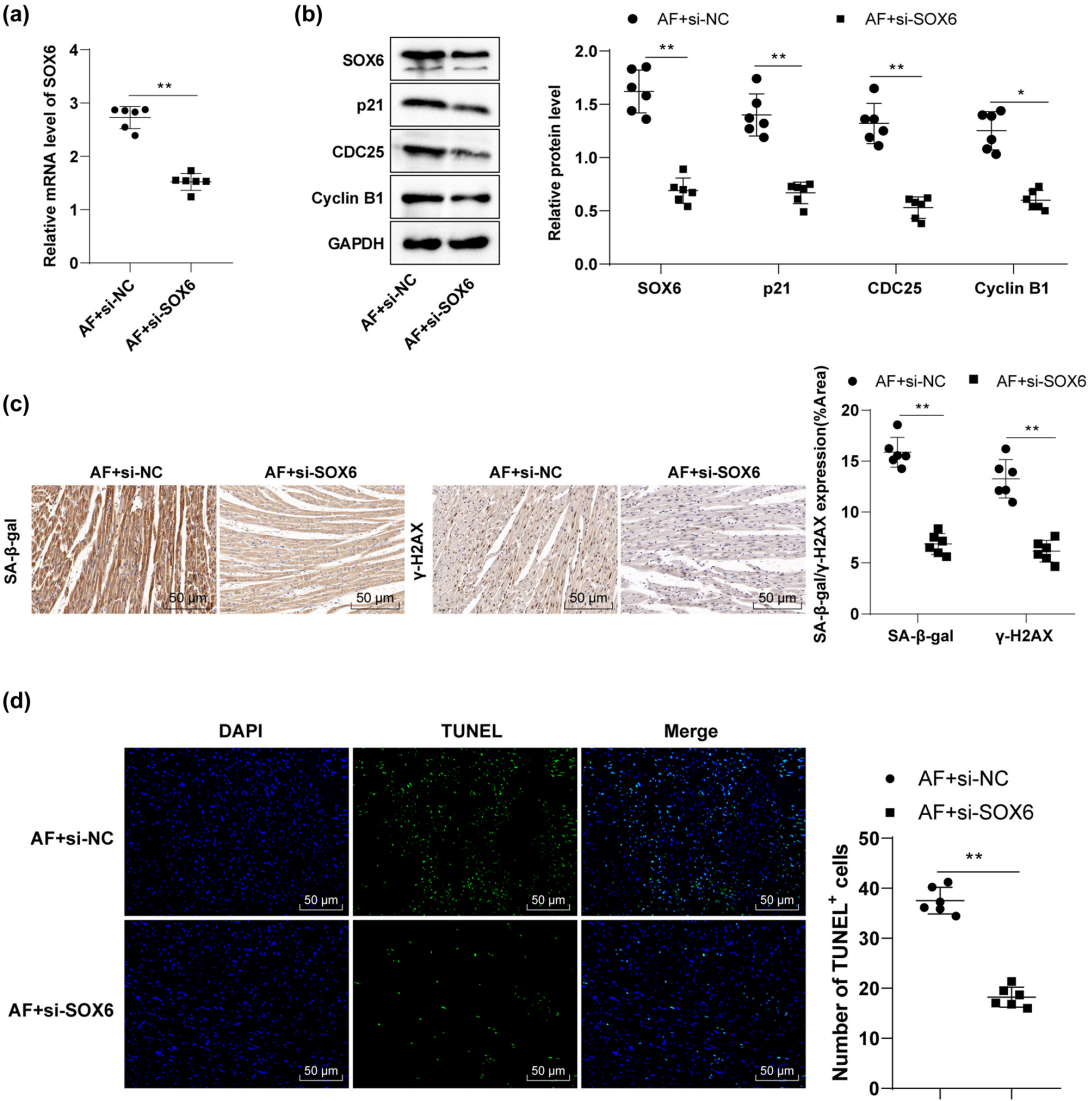
SOX6 silencing downregulated p21 and mitigated cell cycle arrest, senescence, and apoptosis in rat cardiomyocytes. Rats were injected with si-SOX6 plasmids via tail veins on day 14 before AF modeling. (a) RT-qPCR measured SOX6 mRNA level in rat myocardial tissues; (b) Western blotting measured SOX6, p21, CDC25, and Cyclin B1 protein levels in rat myocardial tissues; (c) immunohistochemistry assay determined SA-β-gal and γ-H2AX protein levels; (d) TUNEL staining assessed cell apoptosis. *N* = 6. An independent sample *t*-test was conducted for comparisons between two groups. **p < 0.01.

SOX proteins, key transcription factors for various and frequent diseases during developmental processes, mediate transcriptional activation or activity repression [33] and p21 upregulation [22,23]. To investigate whether SOX6 affects cardiomyocyte behaviors in AF rats by regulating p21 expression, we injected the si-SOX6 plasmids into rats through the tail vein on day 14 before AF modeling. The results unraveled that compared with the AF + si-NC group, the AF + si-SOX6 group exhibited reduced SOX6 levels in myocardial tissues (Figure 5a and b, *p* < 0.01); diminished p21, CDC25, and Cyclin B1 levels (Figure 5b, *p* < 0.01); decreased SA-β-gal and γ-H2AX levels (Figure 5c, *p* < 0.01); and lowered cell apoptosis (Figure 5d, *p* < 0.01). In short, the inhibition of SOX6 expression downregulated p21 and alleviated cardiomyocyte cycle arrest, senescence, and apoptosis in AF rats.

## 4 Discussion

AF, featured by an irregular and commonly rapid heart rate, is a common and fatal arrhythmia, with growing incidences and public health burdens [34]. In particular, fibrosis exerts a causative role in various heart diseases and is a vital contributor to AF episodes [35]. miRNAs are dominantly responsible for manipulating the expression of genes that are involved in cardiac fibrosis, conduction, automaticity, and excitability, thus regulating AF development [36]. Moreover, SOX proteins exert an essential function in normal heart morphogenesis [15]. In this study, our findings demonstrated that miR-499-5p suppressed early cardiomyocyte senescence and atrial fibrosis by targeting SOX6 and downregulating p21, thereby improving AF in rats.

miR-499 is a muscle-specific miRNA that is extensively enriched in cardiac tissues, which can trigger the differentiation of human cardiomyocyte progenitor cells into cardiomyocytes *in vitro* [37]. SOX6, regulated by miRNAs, is a pivotal mediator in adult tissue regeneration, homeostasis, and physiology, and aberrant expression of SOX6 is implicated in diverse diseases, including cardiomyopathy [38]. First, our results revealed downregulated miR-499-5p and upregulated SOX6 in the myocardium of AF rats. Genome-wide RNA-Seq analysis has unveiled the reduction of miR-499-5p in arrhythmogenic cardiomyopathy transgenic hearts, and miR-499-5p contributes to *in vitro* cardiac cell proliferation and differentiation by directly targeting SOX6 [12]. Intriguingly, Trbp manipulates heart function via miRNA-mediated SOX6 repression, and SOX6 overexpression leads to a decrease in cardiac function [17]. The aforementioned evidence supported the involvement of miR-499-5p and SOX6 in AF development.

Compelling evidence suggests that atrial fibrosis is crucial for AF maintenance and perpetuation [39]. α-SMA and TGFβ1 are acknowledged pro-fibrotic biomarkers, and the accumulation of collagen I and collagen III is accountable for enhancing tissue stiffness and causing cardiac diastolic dysfunction [40]. Therefore, we further explored the roles of miR-499-5p in AF, especially in atrial fibrosis. Our results unraveled that miR-499-5p overexpression shortened the AF duration; ameliorated pathological alternations of atrial fibrosis; and reduced levels of collage I, α-SMA, and TGFβ1 in AF rats. Much in line with previous studies [41,42], our findings also highlighted that miR-499-5p targeted SOX6 and SOX6 overexpression counteracted the mitigative effects of miR-499-5p upregulation on atrial fibrosis. Consistently, miR-499-5p alleviates hypoxia/reoxygenation-induced cardiomyocyte injury via targeting SOX6 [43], indicating the cardioprotective effect of miR-499-5p. Although research illustrating the effects of miR-499-5p in atrial fibrosis is limited, among other fibrosis, restoring miR-499-5p decreases collagen fibers and pulmonary fibrosis degree in sepsis-induced lung injury mice by depleting SOX6 [14]. Notably, another SOX protein, SOX9, is capable of facilitating cardiac fibrosis [15]. To sum up, miR-499-5p palliated atrial fibrosis by targeting SOX6, thereby improving AF in rats.

As broadly acknowledged, accelerated senescence can contribute to AF, manifested by increased SA-β-gal activity and elevated p53, p21, and p16 levels in the atrium of AF individuals under 60 years old, and additionally, SA-β-gal activity and p16 are positively associated with the degree of atrial fibrosis [18]. Importantly, cell senescence is considered a stable cell cycle arrest [44], and p21, a vital cyclin-dependent kinase inhibitor, facilitates cell cycle arrest by responding to various stimuli [45]. There is also evidence suggesting that the cardioprotective role of histone deacetylase inhibitors largely relies on the suppression of cardiac fibrosis and regulation of cell cycle arrest, apoptosis, and autophagy [46]. Unsurprisingly, AF rats in our study exhibited increased levels of p21, CDC25, Cyclin B1, SA-β-gal, and γ-H2AX in the myocardium and raised cardiomyocyte apoptosis. However, there is no research investigating the relationship between SOX6 and p21 in AF, and only several studies have unraveled that SOX6 can suppress tumor cell proliferation via upregulation of p21 and augment cell senescence [22,47,48]. Additionally, SOX6 potentiates cardiomyocyte apoptosis through lipopolysaccharide-elicited miR-499 repression [49]. Innovatively, our findings elucidated that SOX6 knockdown reduced p21 levels and attenuated cardiomyocyte cycle arrest, senescence, and apoptosis in AF rats.

To conclude, this study demonstrated that miR-499-5p palliated atrial fibrosis and cardiomyocyte senescence by targeting SOX6 and downregulating p21 in AF rats. The rat has a high degree of physiological similarity to humans and is an ideal choice for many experiments, especially those involving cardiovascular, brain, and spinal cord. The rat genome contains approximately 25,000 genes, 90% of which match those of mice as well as humans. Almost all the human genes associated with diseases find a counterpart in the rat genome, and they appear highly conserved during mammalian evolution. Previous research reveals that miR-499 is a myocardial-specific miRNA and is the most downregulated miRNA in arrhythmogenic cardiomyopathy [12]. Conserved miRNAs play an important regulatory role during development [50], and their target genes are also conserved in vertebrates [51]. Additionally, SOX6 is highly conserved in many mammalian species [52]. To this end, we hypothesized that miR-499-5p and the recognition site of miR-499-5p/SOX are highly conserved in humans and rats. We will verify their conserved nature in future research.

However, there still exist several limitations. The precise molecular mechanism of SOX6 in regulating p21 in myocardial tissues of AF rats remains elusive. Equally importantly, the specific cell phase in which cell cycle arrest occurs during cardiomyocyte senescence warrants in-depth investigation. In addition, this study only explored the mechanism of miR-499-5p/SOX6 in AF at the animal level and did not validate it at the clinical level.
